# Case Report: Rheumatoid arthritis patient with hip joint infection and acetabular protrusion undergoing total hip arthroplasty: a case study and literature review

**DOI:** 10.3389/fsurg.2025.1534279

**Published:** 2025-03-18

**Authors:** Jun Zhou, Weijie Zhou, Peng Wang, Yinwei Zhang, Weitao Chu, Jun Fan, Hui Lu

**Affiliations:** ^1^ Department of Orthopedics, Jinyun People’s Hospital, Lishui, Zhejiang, China; ^2^ Department of Orthopedics, No. 903 Hospital of PLA Joint Logistic Support Force, Hangzhou, Zhejiang, China; ^3^ Department of Orthopedics, The First Affiliated Hospital, Zhejiang University, Hangzhou, Zhejiang, China

**Keywords:** rheumatoid arthritis, hip arthritis, acetabular protrusion syndrome, total hip arthroplasty, hip joint infection, anti-infective therapy

## Abstract

The chronic autoimmune disease rheumatoid arthritis (RA) affects primarily the synovial joints, leading to hip joint deformity and dysfunction manifestations such as acetabular protrusion and joint infection. We present the case of a female patient RA complicated by severe hip arthritis and acetabular protrusion syndrome. The disease progressed rapidly with elevated preoperative inflammatory markers, initially overlooking hip joint infection. The patient underwent total hip arthroplasty, and intraoperative synovial fluid bacterial culture revealed Gram-negative rod bacteria. Aggressive postoperative anti-infective therapy effectively controlled the infection. Therefore, early diagnosis and treatment of infections are particularly important.

## Introduction

Rheumatoid Arthritis (RA) is an autoimmune disorder that primarily affects the synovial joints. It can cause synovial hyperplasia and cartilage destruction, leading to hip joint deformity and dysfunction (1). Acetabular protrusion occurs in 5.2% of patients with RA. Acetabular protrusion refers to the femoral head invagina breaks through the medial wall of the acetabulum and exceeds Kohler's line (the line between the medial edge of the ischium and the medial edge of the ilium) (2). In RA patients, the relentless synovial inflammation and progressive joint destruction contribute to changes in the morphology of the acetabulum, potentially leading to the development of acetabular protrusion. The rate of progression of acetabular protrusion in RA is approximately 2–3 cm a year (3). The implications of this condition are further compounded when complicated by secondary factors, such as joint infection. Infection will accelerate the progress of AR (2). Hip joint infections in RA pose significant challenges due to the compromised joint structure and altered immune responses inherent in the disease (4). Understanding the intricate interplay between RA, acetabular protrusion, and the potential complication of infection is crucial for effective management. This background sets the stage for a comprehensive exploration of a specific case involving a patient with RA, secondary acetabular protrusion, and the added complexity of joint infection, ultimately resulting in rapid acetabular deterioration (5).

The use of antibiotics in bone cement is a common method to prevent and treat infections in orthopedic surgeries. Antibiotics can be directly introduced into implanted materials, such as bone cement, to provide a local high concentration of antibiotics, which can effectively kill bacteria and prevent infection (6). Physiological fluids contamination can significantly affect the mechanical properties of acrylate bone cement. it is important to minimize contamination during the preparation and implantation of bone cement to ensure its effectiveness and safety (7).

In this case study, we present a female patient with RA complicated by severe hip arthritis and acetabular protrusion syndrome, who also developed a hip joint infection. The patient underwent total hip arthroplasty, and intraoperative synovial fluid bacterial culture revealed Gram-negative rod bacteria. Aggressive postoperative anti-infective therapy effectively controlled the infection. This case highlights the importance of early diagnosis and treatment of infections in RA patients, especially those with elevated preoperative inflammatory markers.

## Case presentation

A 58-year-old patient presented to Jinyun People's Hospital with a history of “repeated polyarticular swelling and pain with morning stiffness for over 10 years, accompanied by right hip joint pain for 1 month.” More than a decade ago, the patient experienced recurrent polyarticular swelling and morning stiffness without apparent cause, lasting about 2 h. The pain and swelling were evident in the interphalangeal, metacarpophalangeal, wrist, and ankle joints, symmetrically. Over the past 4 years, there has been a gradual development of deformities in multiple joints of the limbs. Despite seeking help from various hospitals, the patient was provided with conservative treatment involving oral non-steroidal anti-inflammatory medicine, and the episodes of polyarticular swelling and pain persisting. One year ago, the patient received regular anti-RA treatment at the current hospital with “Methotrexate 10 mg once a week, Hydroxychloroquine 0.1 g twice a day, and Leflunomide 25 mg twice a day.” However, the patient suffered from right hip pain caused by a car accident 1 month ago. At that time, there were no obvious signs of fracture in Bilateral Hip Anteroposterior x-ray. Currently, the patient still experiences significant pain in the right hip joint, affecting daily activities and mobility.

**Figure 1:**
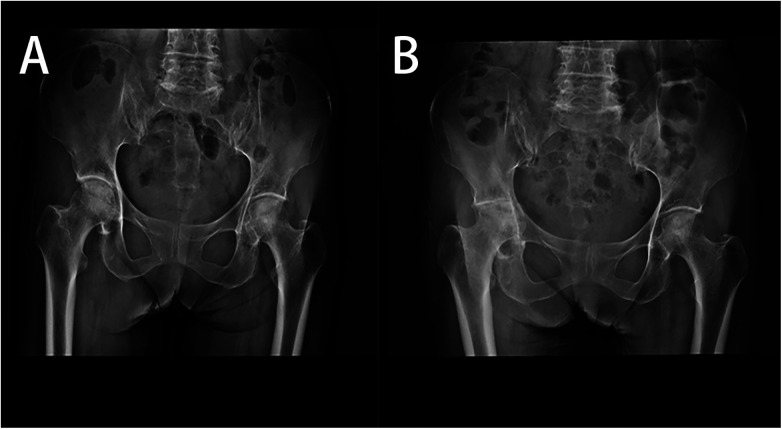
**(A)** 2023-07-14 Bilateral hip anteroposterior x-ray; **(B)** 2023-08-24 bilateral hip anteroposterior x-ray.

Upon admission, the patient had a body temperature of 36.0°C, blood pressure of 105/62 mmHg. On examination: the patient was alert with clear consciousness, and exhibited signs of anemia and emaciation. There were Button deformity and swan-neck deformities in both hands, with some metacarpophalangeal and proximal interphalangeal joints showing spindle-shaped swelling and tenderness. Tenderness was noted in the right sacroiliac joint, and percussion tenderness was present. The right tibiofibular joint had no tenderness, and the “4-sign” test on the right side was positive. Restricted movement was observed in the right lower limb, with no edema in both lower limbs. Muscle strength and tone in all limbs were normal, and Babinski's sign was negative. Preliminary blood tests revealed a white blood cell count of 6.4 × 10^9^/L, 80.0% neutrophils, CRP 51.13 mg/L (normal range <6.00 mg/L), and an ESR 113 mm/h (normal range 0–20 mm/h). A radiological examination on July 14, 2023, showed no acetabular protrusion in the right hip joint (Figure 1A). On August 24, 2023, bilateral hip x-rays indicated rheumatoid arthritis in the right hip joint with acetabular protrusion (Figure 1B).

Preoperatively, the patient had elevated ESR (erythrocyte sedimentation rate) and CRP (C-reactive protein), In the acute stage of rheumatoid arthritis, there may be a rise in ESR and CRP values and there were no signs of infection such as obvious redness and swelling in the hip joint. Therefore, no further testing was performed. On September 14, 2023, a right total hip arthroplasty and bone grafting were performed under general anesthesia. No obvious signs of inflammation or infection were found when the joint capsule was opened during the operation. During the surgery, a definitive diagnosis was established through germiculture.

**Figure 2:**
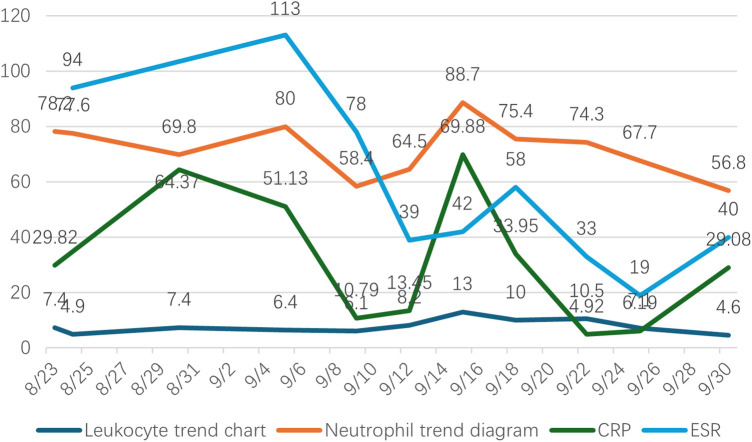
Inflammation trend chart.

**Figure 3:**
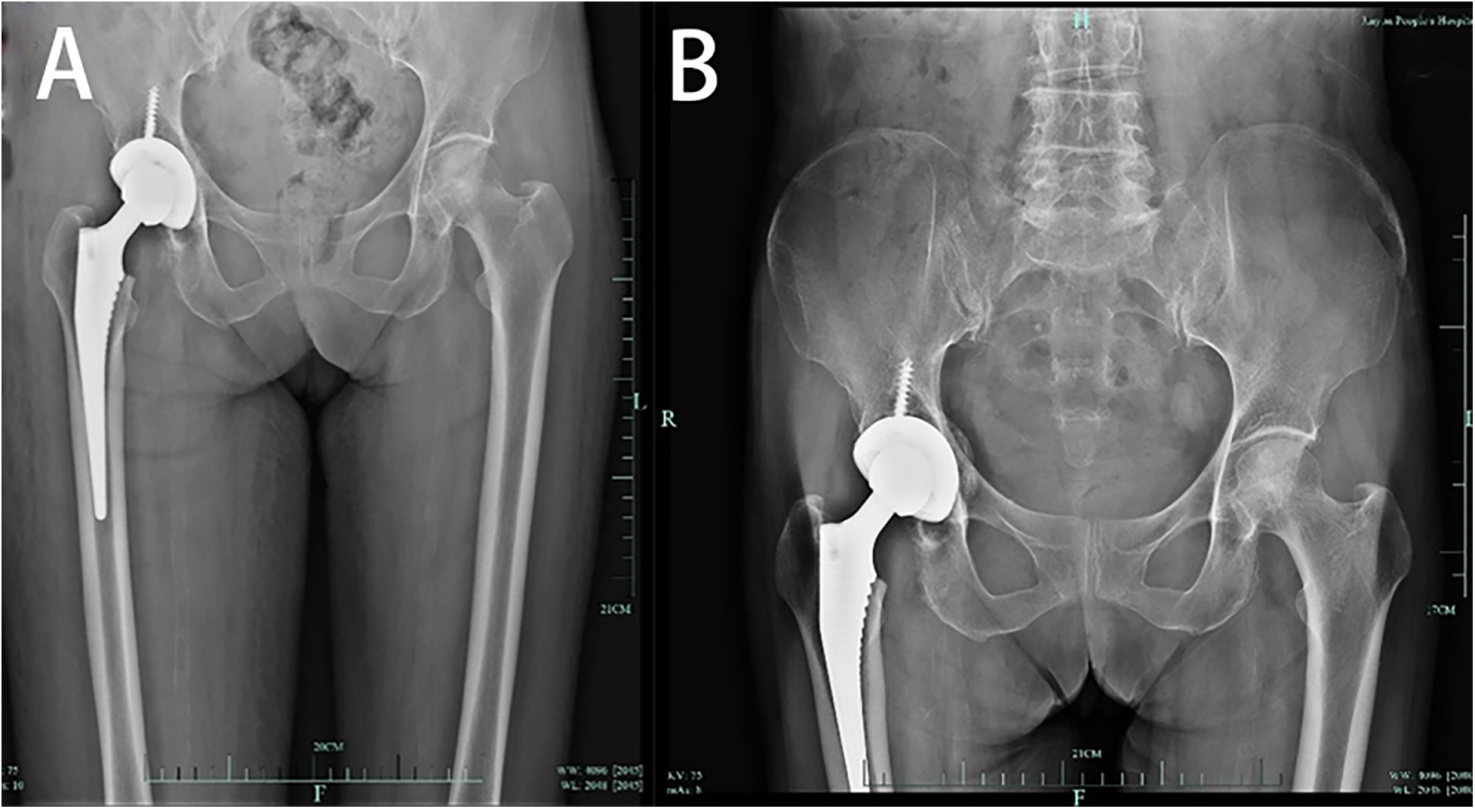
**(A)** X-ray reexamination after operation; **(B)** X-ray was reexamined after 3 months.

Since the possibility of infection could not be ruled out, cefuroxime 1.5 g ivgtt bid was used for anti-infection treatment after operation. The patient's blood tests on the first postoperative day showed an ESR of 42 mm/h, a neutrophil percentage of 88.7%, a CRP level of 69.88 mg/L, and a white blood cell count of 13.0 × 10^9^/L (Figure 2). Culture during the surgery identified Streptococcus constellatus subspecies, which showed sensitivity to vancomycin. Thus, on September 18, 2023, the antibiotic switched to vancomycin 1.0 g ivgtt q12h for anti-infective therapy. On the second postoperative day, drainage tube cultures revealed the presence of Moraxella species, and treatment with vancomycin was continued. After anti-infection treatment, the infection markers decreased obviously, which indicated that the treatment was effective (Figure 3A). After discharge from hospital, moxifloxacin was given 1 tablet once a day to continue anti-infection treatment. Three months after operation, the patient reexamined the x-ray (Figure 3B) and found that the prosthesis was in good position and there was no obvious sign of infection. Patient can walk normally, the hip joint has good mobility, and the pain is obviously relieved.

The patient underwent a comprehensive rehabilitation plan, including physical therapy and rehabilitation exercises, resulting in gradual restoration of joint function. Infection in the patient was effectively controlled, and a favorable outcome was achieved post total hip arthroplasty.

## Discussion

RA is a chronic inflammatory joint disease characterized by persistent synovial inflammation, leading to structural damage within the joints (8). In RA patients, infections of the hip joint can result in progressive destruction of the joint. Clinical manifestations include hip joint pain, swelling, redness, and systemic symptoms such as fever. In studies addressing the heightened risk of infection in RA patients, various factors, including the severity of RA disease, levels of inflammation, and the use of disease-modifying antirheumatic drugs (DMARDs), have been associated with an increased susceptibility to infections (9, 10). Due to the fact that RA can induce arthritis and joint damage, there is an overlap in both symptoms and signs with hip infection, making the diagnosis a challenging task as it may obscure the presentation of symptoms.

Diagnosis relies on a comprehensive evaluation of clinical symptoms, combined with imaging modalities (x-rays, CT, or MRI) and laboratory tests. Patients with RA complicated with hip infection usually show typical symptoms and signs of RA, such as joint pain, swelling, and morning stiffness (11). At the same time, there may be infectious symptoms such as hip pain, progressive joint destruction, fever and chills. ESR and CRP are more reliable infection indicators than WBC. An ESR more than 30 mm/h was used as an indicator of deep infection, with a sensitivity of 97%, specificity of 39%, positive predictive value of 41%, and negative predictive value of 96%. The sensitivity, specificity, negative predictive value, and positive predictive value for CRP values over 10 mg/L were 94%, 71%, 59%, and 96% (12, 13). A serum procalcitonin (PCT) level of 0.5 ng/ml or higher strongly indicates the presence of bacterial infection in patients with RA, and it is advisable to promptly initiate antibiotic treatment for such patients displaying any indications of infection (14). Deep-seated infections can be more accurately diagnosed when ESR and CRP testing are combined. The analysis of frozen sections of total hip replacement showed that the sensitivity was 45% and the specificity was 92% (15). Presepsin is a protein that is significantly high in the blood of individuals with sepsis. Presepsin is regarded a diagnostic and prognostic indication to measure the severity of sepsis and has been found as a biomarker for bacterial illnesses. In severe infections, presepsin may be a better indicator of the severity of infection than PCT (14).

X-ray and CT scan have reference value in the diagnosis of active infection. X-ray and CT scanning can be used to evaluate joint destruction and bone changes (16). MRI is helpful to evaluate osteomyelitis and soft tissue infection. Early detection aids in timely intervention to prevent ongoing deterioration of joint function. In RA patients, the progression of acetabular protrusio may closely correlate with chronic joint inflammation and synovial impact. Septic arthritis requires one of the following four criteria: (a) bacteria isolation from the afflicted joint; (b) solation of the pathogen in the context of septic arthritis from a different source (blood or bodily fluid); (c) typical clinical signs and turbid synovial fluid in a patient without a history of antibiotic treatment; (d) characteristic histopathologic features on postmortem or pathologic examination suggestive of septic arthritis (17). When complicated by infection, the situation becomes more complex as the infection accelerates the process of joint destruction. This scenario may lead to a rapid deterioration of the acetabulum.

Streptococcus constellatus was cultured during operation. Aspiration is the most common risk factor for S. constellatus infection, followed by direct implantation from trauma, surgery, extension by contiguity, and hematogenous dissemination (18). In this patient, MTX and Leflunomide was used to treat RA. Opportunistic infections are potential complications of MTX and Leflunomide treatment, which may occur even when the white blood cell count is within normal range (19). Streptococcus constellatus and Moraxella is conditional pathogens. When the body's resistance is low, the chance of infection is high. When anaerobic bacteria are combined, the toxicity increases (20). Bone destruction progresses very rapidly (21). If appropriate antibiotic therapy is not initiated within 24–48 h, there may be persistent joint dysfunction, cartilage degradation, and subchondral bone loss (22). If the progress is rapid, the possibility of infection must be considered, and whether infection determines the choice of surgical methods. In this case, we observed a combined infection of Streptococcus constellatus and Moraxella, leading to rapid deterioration of the patient's hip joint and resulting in inward collapse of acetabulum.

The study discovered no distinction between the single-stage and two-stage groups' rates of reinfection and reoperation for hip infections (23). One-stage surgery for hip infection requires only one surgical procedure, and reduces the duration of antibiotic treatment, length of hospital stay, and relative overall cost. The outcome of one-stage replacement joint replacement depends on the efficiency with which debridement is performed and biological load is reduced (24). The posterolateral approach is commonly used for the surgery. For acetabular protrusion, the main goal of THA is to restore the natural rotation center (COR) (25). For every 1 mm of native COR undercorrection, there is a 24% chance of aseptic cup loosening (26). Acetabular protrusion is accompanied by a defect or incomplete closure of the medial acetabular wall. Excessive medialization of the COR has the risk of invading the quadrilateral lamina. To cover the medial wall defect and fix the hip's COR, granular autografts from the femoral head, iliac crest, and trochanter were used (25, 27). For older patients (over 65 years) with osteoporosis, the use of bone cement type prostheses may be considered because of their better initial fixation (28, 29). Orthopedic infections can be effectively treated using the implantation of antibiotic-loaded bone cement, and locally released antibiotics can directly raise local antibiotic concentrations at the infection site (6). Following the preventive treatment, the antibiotic regimen was primarily culture-specific. There was a 2-week minimum length for the intravenous treatment. Oral therapy can then be continued depending on the resistance of the infecting organism and the availability of oral agents (30).

In patients with prominent rheumatoid disease, the 15-year survival rate for cemented THA was 85%, which was not statistically different compared to uncemented THA (26). Most patients are able to significantly improve hip function, reduce pain, and improve quality of life after surgery. However, some patients may experience long-term mild pain or limitation of movement, especially if the preoperative hip lesion is severe or combined with other complications. The risk of recurrent infection is a significant concern in patients with a history of hip joint infection, especially in the context of RA. The compromised immune response in RA patients, coupled with the presence of a foreign body (the implant), creates a conducive environment for bacterial colonization and infection. In this case, the patient was treated with a combination of antibiotics and surgical intervention, which effectively controlled the initial infection. However, the risk of recurrence cannot be overlooked. Long-term follow-up and surveillance are essential to monitor for any signs of reinfection. Strategies to minimize the risk of recurrence may include continued use of prophylactic antibiotics, regular monitoring of inflammatory markers, and patient education on infection prevention. The longevity of the implant is another critical aspect to consider. The most common complication of hip arthroplasty is aseptic loosening (25). The stability of the prosthesis depends on the strength of the connection between bone cement and bone tissue. Contamination of physiological fluids can disrupt the polymerization of bone cement, resulting in partial curing, which can have a substantial impact on the mechanical characteristics of bone cement and raise the possibility of prosthesis loosening (7). The addition of antibiotics to bone cement has been shown to have a minimal impact on the mechanical properties of the cement, as long as the antibiotic concentration is within a certain range. However, excessive amounts of antibiotics can affect the polymerization reaction and curing process of the bone cement, leading to a decrease in strength. Therefore, it is crucial to balance the antibiotic concentration to ensure both effective infection control and sufficient mechanical strength of the bone cement (31). The infection rate has remained at around 2.6% since the 1990s and appears to be 2–6 times higher than in the general population (32). Key risk factors include incomplete eradication of infection during initial treatment, biofilm formation on implants, and the presence of polymicrobial or antibiotic-resistant infections. Regular monitoring of infection markers, such as CRP, ESR, PCT, or presepsin, is important for early detection of latent or recurrent infections (14). Detailed postoperative evaluation of implant stability with advanced imaging modalities such as MRI or CT and early detection of complications are recommended.

Treatment for such cases necessitates a comprehensive approach, considering RA management, correction of acetabular protrusio, and infection control. A comprehensive treatment plan should be made according to the specific situation of RA patients combined with acetabular invagination and infection. The treatment usually includes antibiotic therapy, surgical drainage and artificial hip replacement, but the optimal choice of surgical strategy hinges on the type of the infection (4). In addition to having a history of infection, patients with static SA do not exhibit any clinical, biochemical, or radiological symptoms of a localized current infection (33). For dormant infections, it is advisable to option for one-stage arthroplasty rather than the two-stage procedure intended for individuals with active infections during arthroplasty (4). Antibiotic therapy can control infection and prevent it from spreading further. Surgical drainage can clear the infection focus and relieve pain and inflammation. Artificial hip replacement can rebuild hip joint function, relieve pain and improve patients' quality of life (34).

The limitation of this case study is the absence of advanced imaging techniques such as MRI or CT scans, which could have provided more detailed insights into the hip joint condition and the extent of acetabular protrusion. These imaging modalities are particularly useful in assessing soft tissue structures and detecting early signs of joint infection or inflammation that standard x-rays may overlook. PCT is a sensitive marker of bacterial infection, and its level changes can more accurately reflect the activity of infection. The lack of PCT detection may make it difficult for doctors to distinguish between inflammation caused by RA itself and inflammation caused by bacterial infection, affecting the accurate judgment of disease progression.

## Conclusion

This case emphasizes the crucial importance of discovering hip joint infection, especially in RA patients with elevated preoperative inflammatory markers. Comprehensive treatment, accurate diagnosis, and aggressive anti-infective therapy are pivotal in successfully managing such complications. A Surgical teams should exercise heightened caution, especially with rapidly progressing conditions and elevated inflammatory markers. Delayed identification and management of suppurative arthritis can result in lasting morbidity and mortality. In cases of suspected infection, intraoperative bacterial culture aids quick diagnosis, enabling targeted postoperative treatment. Proactive postoperative anti-infective therapy is vital for infection control, joint stability, and patient recovery. Recognizing infection as a potential complication is key when dealing with complex cases, contributing to better surgical outcomes and patient recovery.

## Data Availability

The original contributions presented in the study are included in the article/Supplementary Material, further inquiries can be directed to the corresponding author.
